# Good mid-term results with the trident peripheral self-locking cup: a clinical evaluation and migration measurement with EBRA

**DOI:** 10.1007/s00402-020-03639-5

**Published:** 2020-11-09

**Authors:** Dietmar Dammerer, Philipp Blum, David Putzer, Andreas Tscholl, Michael C. Liebensteiner, Martin Thaler

**Affiliations:** 1 Department of Orthopaedics and Traumatology, Medical University of Innsbruck, Anichstrasse 35, 6020 Innsbruck, Austria; 2 Department of Experimental Orthopedics, Medical University of Innsbruck, Sonnenburgstr. 16, 6020 Innsbruck, Austria

**Keywords:** Cup migration, Total hip arthroplasty, Cementless, Einzel–Bild–Röntgen–Analyse (EBRA)

## Abstract

**Introduction:**

The most common cause of failure in total hip arthroplasty (THA) is aseptic loosening. Uncemented cup migration analysis by means of Einzel–Bild–Roentgen–Analyse (EBRA) has shown to be a good predictive indicator for early implant failure if the cup migrates more than 2 mm within 4 years after surgery. In this study, we performed a migration analysis of an uncemented peripheral self-locking (PSL) press-fit cup after 4 years follow-up.

**Materials and methods:**

We retrospectively reviewed all patients who received a trident PSL press-fit cup at our department between 2004 and 2017. A total of 636 patients were identified. As inclusion criteria for radiological analysis, a minimum follow-up of 2 years was defined. We reviewed medical histories and performed radiological analysis using EBRA software. EBRA measurements and statistical investigations were performed by two independent investigators.

**Results:**

A total of 149 cups in 146 patients (female 82; male 64) met our inclusion criteria. Mean age at surgery was 65 years (33–89). We found a significant improvement in the WOMAC score pre- to postoperative (*p* < 0.0001). EBRA migration analysis showed a mean total migration of 0.6 mm (0.0–8.2) over our follow-up period of 4 years. Of the investigated cups, 69.8% showed a migration rate smaller than 2 mm in the investigated follow-up.

**Conclusion:**

The acetabular cup used in our study provides low migration at final follow-up. Therefore, a good long-term outcome can be expected for the PSL cup.

**Trial registration:**

Trial registration number is 20181024-1875 and date of registration is 2018-10-24.

## Introduction

The most common cause of failure in total hip arthroplasty (THA) is aseptic loosening [1]. According to the literature, a cup migration of more than 1 mm within the first 2 years, or more than 2 mm at 4 years after surgery is a well-established risk factor for early implant failure [2–5]. Previously published studies of uncemented cup migration by Einzel–Bild–Roentgen–Analyse (EBRA) have shown this to be a good predictive indicator and threshold for later aseptic loosening [6–8].

Einzel–Bild–Roentgen–Analyse is a computer-assisted method for measuring the migration of acetabular cups using standard anterior–posterior (ap) pelvic radiographs without requiring additional means at exposure (e.g. ball markers) [9]. EBRA has a proven accuracy and sensitivity in detecting migration of more than 1 mm as compared to RSA (roentgen stereophotogrammetric analysis) [10, 11].

As THA is one of the most successful medical procedures, various implant designs are available. Peripheral self-locking designs are commonly used as an acetabular implant because the rationale behind the PSL design is to provide better primary stability at the outer acetabular rim. The cup used in this study is the Trident PSL (peripheral self-locking) by Stryker^®^ (Stryker, Kalamazoo, Michigan, USA). According to the Australian Orthopaedic Association National Joint Replacement Registry (AOANJRR), with a total of 8450 implanted cups Trident was the most common cup used in primary total conventional hip replacement in 2018, whereby AOANJRR does not exactly distinguish between PSL and hemispherical cup type [12]. Since the Trident cup system was released in 1999 [13], it has been widely used for primary as well as revision THA. The Trident peripheral self-locking cup has a 1.8-mm peripheral press-fit built into the shell [14]. This design is intended to allow better fixation to the peripheral lunate of the acetabulum [13]. In 2012, Nunag et al. presented preliminary data from a migration analysis of the Trident PSL with a very low number of cases. Nunag et al. [13] mentioned good clinical function of the implant, whereby migration analysis showed radiological instability in most implants within the first 2 years after surgery.

In the present study, we investigated the clinical results and migration behavior using EBRA of the uncemented Trident PSL cup with a mean follow-up of 4 years.

## Materials and methods

After approval by the local ethics committee (Medical University of Innsbruck, Austria, Europe), we applied a retrospective study design and reviewed all consecutive patients who had received an uncemented Trident PSL cup in our Department between 2004 and 2017. During this time, a total of 636 Trident PSL acetabular cups were implanted as primary THA.

We investigated the medical histories for sociodemographic data, surgical approach, body mass index, cut-to-suture time, and the indication for THA. In addition, the Western Ontario and McMaster Universities Osteoarthritis (WOMAC) Index was utilized pre- and one year postoperatively as patient-reported outcome measure for function and pain [16].

**Fig. 1:**
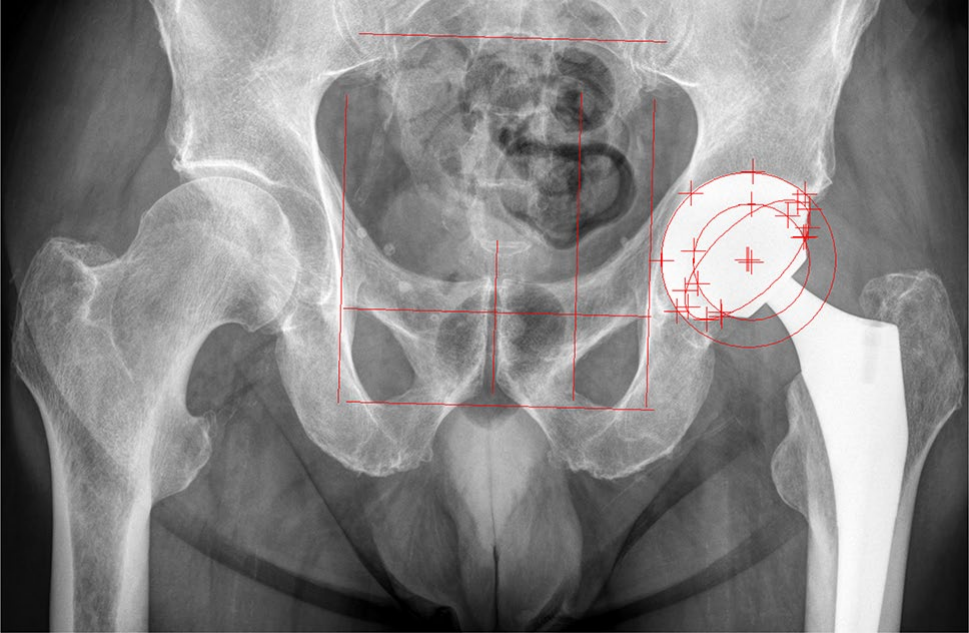
Postoperative anterior–posterior x-ray of a primary total hip arthroplasty with EBRA cup software lines included

**Fig. 2:**
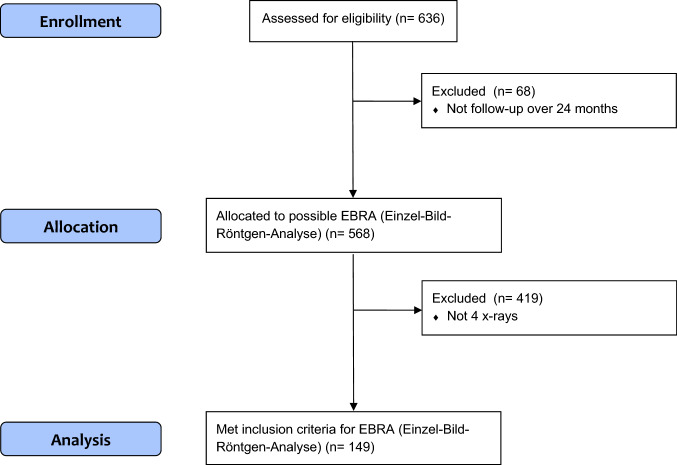
CONSORT guidelines cup flow diagram

Prosthetic stability and cup migration were assessed with EBRA (German: Einzel–Bild–Röntgen–Analyse) [9] from plain x rays. EBRA is a well-established method for evaluating standard anterior–posterior radiographs without requiring additional means at exposure (e.g. ball markers) (Fig. 1). Simulating the spatial situation, it computes parameters of longitudinal and transverse migration of prosthetic cup and femoral head. Migration of the femoral head, the acetabular cup and the wear in the horizontal and vertical directions can be studied. A comparability algorithm using a grid of transverse and longitudinal tangents of the pelvis contour divides serial radiographs into sets of comparable ones. Migration is measured only between comparable radiographs. The 95% confidence limits for EBRA results are 1.0 mm for longitudinal and 0.8 mm for transverse migration [9]. All radiographs were taken in a strictly standardized manner according to the EBRA protocol (anterior–posterior radiographs; patient standing in an upright position; full weight-bearing) [9]. In our department we follow patients with radiographs routinely before discharge, 6 weeks after surgery, 12 months postoperative and at a consecutive annual check-up. In addition to this routine, we perform additional radiographs if the patient has any complaints, sorrows or questions concerning the THA**.** In our study, migration analysis with EBRA called for a minimum of four radiographs per patient and a minimum radiological follow-up of 2 years. Cup migration analysis was performed by one independent investigator, who was not involved in the surgeries or postoperative patient treatment. All patients in the study group fulfilled the EBRA criteria. The head and cup sizes used for EBRA calibration were taken from the intraoperative protocol. Cup flowchart is shown as Fig. 2.

In addition, as suggested in the literature, our patient cohort was split into two groups to measure the cup size effect: patients with an implanted cup size  ≥ 54 mm and patients with a cup size  < 54 mm [15, 16].

## Statistics

Mean, median, range, and standard deviation were calculated for the various measurement parameters. For the analysis, Access and Excel (Microsoft Office Professional Plus 2010, Redmond, WA, USA) as well as Graph Pad Prism (Version 8.0, GraphPad Software, Inc., La Jolla, CA, USA) were used. WOMAC scores were compared using the Wilcoxon’s signed-rank test. Total migration was calculated with the Pythagorean theorem expressing the length of the vector. We defined loosening as a total migration of more than 2 mm within 4 years [6]. For comparison of the total migration divided by cup size the non-parametric Mann–Whitney *U* test was used. A *p* value of 0.05 was considered statistically significant.

## Results

A total of 149 cups in 146 patients (female 82; male 64) fulfilled our inclusion criteria. In three patients the Trident PSL cup was implanted on both sides. At the time of surgery, the patient mean age was 65 years (33–89) and mean body mass index was 26.8 kg/m^2^ (15.2–39.4). Mean follow-up was 4 years (2.0–9.8). Pre-operative diagnosis was osteoarthritis in 130 hips (87.3%), avascular necrosis of the femoral head in nine (6.0%), hip dysplasia and secondary osteoarthritis in nine (6.0%) and femoral neck fracture in one hip (0.7%). Mean cut-to-suture time was 72 min (33–188). The cup sizes most frequently used in our study were 52 mm (17.4%) and 56 mm (17.4%). The mean cup inclination was 42.3° (24.1°–61.3°) and the mean cup anteversion was 18.5° (4.3°–48.5°). Further details are shown in Tables 1 and 2.

**Table 1:** Demographics of study patients

Number of patients	
Female	82
Male	64
Total	146
Mean age (years)	65 (33–89)
BMI (kg/m^2^)	26.8 (15.2–39.4)
Cut-to-suture time (min)	72 (33–188)
Surgical approach	
Direct anterior approach	146
Direct anterior approach with release of the musculus tensor fasciae latae	1
Missing data	2
Surgical position	
Supine	149
Preoperative diagnosis	
Osteoarthritis	130
Avascular necrosis of the femoral head	9
Hip dysplasia	9
Fractures of the femoral neck	1

Range is given in brackets

**Table 2:** Details of implanted components

Trident PSL cup size (mm) (%)	
46	3 (2.0)
48	8 (5.4)
50	28 (18.8)
52	26 (17.4)
54	25 (16.8)
56	26 (17.4)
58	22 (14.8)
60	4 (2.7)
62	6 (4.0)
64	1 (0.7)
Head size (mm) (%)	
28	30 (20.1)
32	100 (67.1)
s36	19 (12.8)

We found a significant improvement in total WOMAC score from 57 (4–99) pre-operatively to 17 (0–95) 1 year postoperatively (*p* < 0.0001). No further significant differences were found for the subgroups (stiffness, etc.) of the WOMAC score.

Migration analysis at 4 years follow-up was calculated for 43 of the 149 cups with an EBRA given comparability limit of 3.0 mm (95% CI). Negative horizontal migration values were defined as medial migration. Negative vertical migration (distal migration) up to 1 mm was caused by the limited accuracy of the EBRA measurement method. A complete set of radiographs at every point of time (e.g. 1–2 years, etc.) was not available for each cup in our study. This resulted in a different number of cases at the corresponding migration behavior analysis over time.

**Fig. 3:**
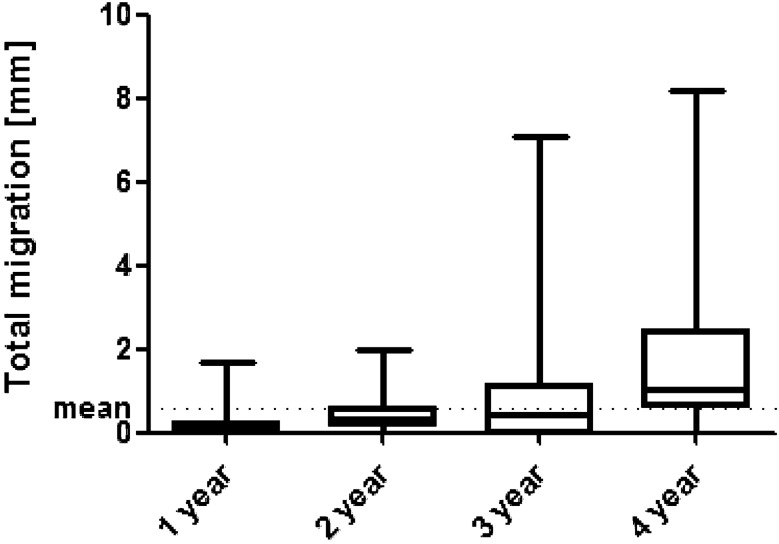
Boxplots showing mean migration and bars showing the minimum and maximum migration for radiological follow-up

Based on Krismer et al.’s [6] definition of aseptic loosening, seven (8.1%) of 86 cups showed a migration of more than 1 mm after 2 years. Of the 149 cups, 43 had sufficient EBRA follow-up to determine migration behavior after 4 years. Of these 43 cups, 13 (30%) migrated more than 2 mm. We found that nearly 70% of the investigated cups showed a migration rate smaller than 2 mm in the investigated follow-up period of 4 years. Table 3 and Fig. 3 show details of migration behavior after 1–4 years. Percentages of migrated cups are given in Table 4.

**Table 3:** Mean total migration in millimetres (mm) over time

	12 months (*n* = 137)	24 months (*n* = 86)	36 months (*n* = 108)	48 months (*n* = 43)
Migration of the trident PSL (mm/year)	0.1 (0.0–1.7)	0.4 (0.0–2.0)	0.8 (0.0–1.1)	1.5 (0.0–8.2)

Range is given in brackets

**Table 4:** Total migration in millimetres (mm) over time

	1 year (*n* = 137)	2 years (*n* = 86)	3 years (*n* = 108)	4 years (*n* = 43)
≤ 1	135 (98.5%)	79 (91.9%)	76 (70.4%)	20 (46.5%)
> 1	2 (1.5%)	6 (7.0%)	21 (19.4%)	10 (23.3%)
> 2	0 (0.0%)	1 (1.1%)	5 (4.6%)	10 (23.3%)
> 3	0 (0.0%)	0 (0.0%)	3 (2.8%)	1 (2.3%)
> 4	0 (0.0%)	0 (0.0%)	1 (0.9%)	0 (0.0%)
> 5	0 (0.0%)	0 (0.0%)	2 (1.9%)	2 (4.6%)

While investigating the cup size effect, we found no statistically significant difference in total migration between the two sub-cohorts: cup size  < 54 and  ≥ 54 mm at 1 year (*p* = 0.9672), 2 years (*p* = 0.1830), 3 years (*p* = 0.4199) and 4 years (*p* = 0.2379) radiological follow-up.

During our follow-up period, four cups had to be revised. All four were due to a periprosthetic infection after 27, 34, 46 and 90 months. No further revisions were required. Six patients died during our clinical follow-up, but not because of surgical or postoperative reasons.

## Discussion

To the best of our knowledge, the present study includes the longest follow-up period and the largest number of uncemented peripheral self-locking cups investigated by means of EBRA to date. As THA is one of the most successful medical procedures, various implant designs are available. Peripheral self-locking designs are commonly used as acetabular implant because the rationale behind the PSL design is to provide better primary stability at the outer acetabular rim. We found the investigated cup to have a very low mean total migration of 0.6 mm/year during our follow-up period of 4 years. Previously published and applied threshold values for cup loosening show that our results are well in line with the current literature. In our study, nearly 70% of the cups showed migration to be less than 2 mm within 4 years. From previously published data we can expect the Trident-PSL cup to have excellent long-term survival.

It is assumed that primary stability is a prerequisite for bony ingrowth of the acetabular component. For this purpose, EBRA is a suitable method for identifying and measuring the migration behavior of THA components [9, 17, 18]. While roentgen stereometric analysis (RSA) is considered the gold standard for migration measurements, Abrahams et al. [10] recently reported good agreement between EBRA cup and RSA measurements of migration of acetabular cups in THA. Knowing that and applying a retrospective study design, we used EBRA cup software for our investigations of migration behavior.

A study by Nunag et al. [13] gives a mean total migration of 1.5 mm for the Trident PSL cup after 2 years follow-up. Assuming the above-mentioned threshold for significant migration of an uncemented cup, the authors stated in their study that 54% of their cups migrated more than 1 mm and another 23% more than 2 mm in the 2-year follow-up [13]. In comparison to the results reported by Nunag et al., we found in our study a significantly smaller rate of cup migration. Our results show that 8.1% of the investigated cups migrated more than 1 mm in 2 years. While in our study 13 (30.2%) cups migrated more than 2 mm after 4 years, only 7 (16.3%) of these cups showed a further migration of more than 1 mm compared to the previous measurement. Although our EBRA migration analysis covered a period of 4 years after surgery, as compared with Nunag’s 2-year follow-up period [13], our mean migration rate of 1.5 mm at 4 years was similar the rate published by Nunag et al. [13]. The patient sample studied by Nunag et al. was smaller, showed a higher cup migration rate and a mean observed radiolucent gap of 1.4 mm between cup and bone in two-thirds of all investigated cases [13]. In contrast to the surgical technique published by the manufacturer, Nunag et al. reported that the actually reamed cavity was on average 1 mm smaller than the reamer [13]. The instruction manual for the surgical technique recommends reaming line to line.

Several factors and the influence of migration were reported in previous studies. Especially the effect of cup size on migration yielded different results. While Stihsen et al. found a significantly greater migration for cups  ≥ 54 mm, Takatori et al. reported a negative correlation between cup size and distance of migration [15, 16]. Stihsen et al. [15] suspected that the soft bone surrounding the larger cup provides a bigger surface for movement, which may lead to increased migration. As we found no statistical differences in total migration between cup size  < 54 and  ≥ 54 mm at 1–4 years radiological follow-up, we can confirm the findings of Stoeckl et al. [19], namely that there is no correlation between cup size and migration. Stoeckl et al. investigated and published the migration behaviour of the Duraloc cup ‘100 Series’ (DePuy Synthes, Warsaw, IN, USA) after 2 and 4 years follow-up. Using the same criteria for migration analysis, 48% of the Duraloc cups showed significant migration and a mean total migration rate of 1.13 mm at 2 years [19]. However, the reduction in migration speed after 4 years observed by Stoeckl et al. [20] provided a better result for the Duraloc cup than the initially surveyed migration rate after 2 years had suggested.

This study has several limitations including the retrospective methodology and the absence of a control group. In addition, other factors that might influence migration behavior of the cup, such as comorbidities and under-, line-to-line- or over-reaming, were not investigated. Furthermore, only 149 (23.4%) of 636 implanted cups were eligible for this study due to a lack of follow-up or insufficient number of x-rays. This may increase the susceptibility of the study to selection bias. Moreover, there was a varying number of radiographs and duration of follow-up for each hip. This may have influenced the migration results due to the smoothing function of the software. Based on the published EBRA-cup threshold by Krismer et al. [6], none of our cups have been revised during the observed follow-up period. Therefore, long-term results are necessary to observe the performance and survival of the investigated cup. Additionally, further studies are needed to state more clearly the relevance of the EBRA-cup threshold by Krismer et al. [6], in short- to mid-term migration behavior studies. Finally, the EBRA cup software uses the horizontal line that can be labeled on the pubic symphysis or the ischial foramen as the reference segment for proximal translation, while assuming that the pelvis is in continuity and is a single reference segment.

In conclusion, we show a low migration rate for a peripheral self-locking cup with excellent clinical results up to 4 years after surgery. From the results of the migration analysis, we can predict good long-term survival for the cup. Further investigation is needed for cups that showed a migration of more than 2 mm at 4 years follow-up.

## Data Availability

Data will be sent if necessary.
